# Prospects for the diagnosis and treatment of sarcopenia in the Philippines

**DOI:** 10.3389/fmed.2024.1501501

**Published:** 2025-01-07

**Authors:** Ji Sun, Weixin Zhang, Peipei Han

**Affiliations:** ^1^Collaborative Innovation Center for Biomedicines, Shanghai University of Medicine and Health Sciences, Shanghai, China; ^2^College of Nursing and Allied Health Sciences, St. Paul University Manila, Manila, Philippines; ^3^College of Rehabilitation Sciences, Shanghai University of Medicine and Health Sciences, Shanghai, China

**Keywords:** sarcopenia, Philippines, rehabilitation, older people, diagnosis

## Abstract

Over the past decade, the elderly Filipino population has significantly increased, rising from 4.6 million seniors, which was approximately 6% of the total population in 2000, to 6.5 million, or around 6.9% of the population in 2010. Projections suggest that by 2030, the percentage of the population aged 60 and above will increase to between 10 and 19%, indicating a significant demographic shift toward an aging population. This shift from a predominantly young population to a rapidly growing elderly demographic underscores the urgent need for effective health promotion and management programs targeting older adults. Sarcopenia, a muscle-wasting disorder, represents a significant global health challenge, particularly affecting the elderly. In the Philippines, the impact of sarcopenia is expected to become more pronounced, potentially straining both individuals and the national healthcare system over the next 15 years, despite the country’s relatively younger overall population. Despite the expanding research on sarcopenia in the Philippines, there remains an urgent need to raise awareness and implement proactive measures to address this escalating health issue.

## 1 Current state of research in the Philippines

The demographic shift toward aging populations in developing countries has intensified the prevalence of sarcopenia, creating unique challenges for healthcare systems already limited in resources (1). In regions like Southeast Asia, Latin America, and sub-Saharan Africa, life expectancy has risen steadily, leading to larger elderly populations than ever before. However, unlike in high-income countries, healthcare infrastructure in many lower- and middle-income nations often lacks adequate diagnostic facilities and trained personnel to address age-related conditions effectively (2, 3). This gap in healthcare capacity leaves older adults at greater risk for untreated muscle degeneration, which can lead to increased disability, decreased quality of life, and a greater financial burden on families and communities (4). The rising burden of sarcopenia in these regions underscores an urgent need for scalable, cost-effective interventions that consider local constraints.

Research employing the Asian Working Group for Sarcopenia (AWGS) criteria has revealed a prevalence of 27.7%, while the revised European consensus (EWGSOP2) reported prevalence rates of 18.18%. A study from a tertiary hospital revealed a higher prevalence of 73.33% (5). Collectively, these findings suggest that sarcopenia is prevalent among Filipino adults aged 60 and older, influenced by various sociodemographic and physiological factors. Current research identifies age, waist circumference, smoking, and alcohol consumption as significant predictors of sarcopenia (6). Sarcopenia is closely associated with reduced physical function and diminished quality of life, underscoring the necessity for national guidelines to address this pressing issue. At the March 2023 Asia-Pacific conference on muscle health and sarcopenia, a multinational panel, including Filipino experts, proposed five core pillars: awareness, education, initiatives, engagement, and research. These pillars are designed to address the gaps and challenges in implementing muscle health assessments and management within clinical practice (7). Additionally, the country launched its first longitudinal study on health and aging (LSHAP) in 2018, using a nationally representative sample and recommending further research on sarcopenia and healthy life expectancy to enhance understanding of the health characteristics of Filipino older adults. A review of studies on sarcopenia in Filipinos aged 60 and above conducted between 2013 and 2023 revealed that most research has been hospital-based and involved patients with comorbidities. Sarcopenia remains inadequately integrated into routine clinical practice. The Geriatric Health-Research Institute (NCGH-RI) has incorporated sarcopenia screening into the Comprehensive Geriatric Assessment (CGA), emphasizing the need for further research and implementation to address this escalating public health concern. Table 1 summarized sarcopenia-related research conducted in the Philippines.

**Table 1:** Summary of sarcopenia-related research conducted in the Philippines.

References	Diagnostic criteria	Type of study	Population	Outcomes
Cruz et al. (51)	General health assessment focusing on functional capacity, mobility, and disability in elderly populations	Longitudinal study	Older Filipinos, unspecified age range, focusing on aging demographics	Higher prevalence of inactivity and functional disability among elderly Filipinos; implications for public health policies
Tee and Tee (26)	EWGSOP criteria with Filipino normative values for muscle mass, grip strength, and gait speed	Cross-sectional study	Healthy Filipino adults aged 20–40, serving as a normative baseline population	Establishment of reference values for muscle mass, strength, and gait speed tailored to the Filipino population
Gabat et al. (6)	EWGSOP definition using Filipino-specific cutoffs for BMI, WC, muscle mass, grip strength, and physical performance	Cross-sectional study	Filipinos aged over 40 years, sampled at an outpatient department of a general hospital	Findings indicate no significant association between obesity and sarcopenia; age, smoking, alcohol consumption, and lower WC identified as significant predictors
Gay-As et al. (5)	EWGSOP and AWGS criteria with multiple normative references for diverse Filipino populations	Scoping review	Older Filipinos aged 60 and above, encompassing both hospital and community settings	Mapping of prevalence, risk factors, and health impacts of sarcopenia among hospitalized older adults in the Philippines

EWGSOP, European working group on sarcopenia in older people; AWGS, Asian working group for sarcopenia; BMI, Body mass index; WC, Waist circumference.

## 2 Mechanism

Sarcopenia is a complex muscle disorder characterized by the progressive loss of muscle mass, strength, and function, predominantly affecting older adults (8). This condition is influenced by a combination of genetic, biological, environmental, and lifestyle factors. The pathogenesis of sarcopenia is multifactorial, involving a combination of genetic predisposition, biological processes, environmental influences, and lifestyle factors. Research has shown that various signaling pathways, such as those involving protein metabolism, muscle regeneration, and inflammation, are crucial in the development of sarcopenia (9, 10). Aging results in the atrophy of fast-twitch type II muscle fibers, which are essential for generating force and power. This leads to a reduction in muscle size and strength, particularly in response to diminished satellite cell activity and impaired muscle regeneration (8). Concurrently, muscle protein synthesis declines with age, exacerbating the negative protein balance. This decline is partly due to reduced activity of the mTOR signaling pathway, which plays a key role in muscle protein synthesis and growth (9, 10). Additionally, aging promotes an increase in muscle protein breakdown, which is further exacerbated by chronic inflammation, oxidative stress, and physical inactivity. This process is mediated by upregulated proteolytic pathways, such as the ubiquitin-proteasome system and autophagy (11, 12). Hormonal changes, including declines in testosterone, growth hormone, and insulin-like growth factor 1 (IGF-1), further exacerbate the loss of muscle mass and strength. These hormonal alterations impair muscle regeneration and increase protein catabolism (13). Chronic low-grade inflammation, a hallmark of aging, is associated with sarcopenia. Pro-inflammatory cytokines, such as interleukin-6 (IL-6) and tumor necrosis factor-alpha (TNF-*α*), play a crucial role in muscle wasting by increasing muscle protein breakdown and inhibiting muscle protein synthesis. Inflammation also disrupts the balance of muscle repair and regeneration (14, 15). Physical inactivity is one of the most significant accelerators of sarcopenia, driving the rapid decline in muscle mass, strength, and functionality. It results in decreased muscle protein synthesis, impaired mitochondrial function, and muscle fiber atrophy, all of which contribute to the progression of sarcopenia (16, 17).

## 3 Diagnosis

The European Working Group on Sarcopenia in Older People (EWGSOP) and the Asian Working Group for Sarcopenia (AWGS) have established diagnostic algorithms for sarcopenia that include assessments of muscle mass, strength, and physical performance (18, 19). These algorithms are widely applied in clinical and research settings, including in countries like the Philippines, where sarcopenia research is becoming more prevalent. In EWGSOP1, the criteria for diagnosing sarcopenia include low muscle mass in conjunction with either low muscle strength or low physical performance (20), with muscle mass being the primary component of sarcopenia. These criteria have been widely studied in European and Asian populations, but there is a need to determine whether these criteria are appropriate for Filipino populations, considering potential ethnic and environmental differences. EWGSOP2, however, prioritizes muscle strength, defining sarcopenia as low muscle strength combined with low muscle quantity or low muscle quality (19), where muscle strength becomes the predominant component. This approach may need modification when applied to the Filipino population, as studies suggest variations in muscle strength and mass across ethnic groups. AWGS2019 also emphasizes the importance of muscle strength. Consequently, these differing criteria may result in variations in the reported prevalence of sarcopenia. A systematic review indicates that the prevalence of sarcopenia in older adults is lower when diagnosed using EWGSOP2 compared to EWGSOP1 (21). A similar trend has been observed in some studies conducted in Southeast Asia, but further research is needed to apply these findings to the Filipino population. A comparative study of sarcopenia prevalence in a large multinational European population sample using various diagnostic criteria (22) revealed similar findings, suggesting that prevalence estimates based on EWGSOP1 tend to be higher than those based on EWGSOP2. However, AWGS2 criteria show a similarly lower prevalence to EWGSOP2, likely due to their shared reliance on muscle mass and muscle strength. Baumgartner et al. (23) emphasized the significance of employing “population-specific diagnostic criteria” for sarcopenia. This is particularly relevant for the Filipino population, where unique genetic, dietary, and environmental factors may influence sarcopenia characteristics. They delineated a methodology for estimating the prevalence of “deficient” relative muscle mass, or sarcopenia, in population-based studies. Consequently, numerous sarcopenia criteria have incorporated the concept of “relative muscle mass.” Therefore, identifying suitable measurement tools and establishing cut-off values in the Philippines that align with international standards will enable researchers and policymakers to adapt actions and recommendations as proposed by organizations such as the EWGSOP.

Dual-energy X-ray absorptiometry (DXA) is a widely accepted and reliable method for assessing muscle mass in older adults and is endorsed by international organizations for diagnosing sarcopenia. However, the applicability of DXA in the Filipino population, due to differences in body composition, requires further validation. Bioelectrical impedance analysis (BIA) provides a portable, non-invasive, and cost-effective alternative to DXA. Recent studies have confirmed BIA as a dependable tool for diagnosing sarcopenia in older adults (23). Handgrip strength serves as a straightforward and cost-effective screening tool for muscle strength. Decreased handgrip strength is a significant predictor of adverse outcomes in older adults, including disability, falls, and mortality (24). Physical performance, especially gait speed, is another crucial diagnostic criterion for sarcopenia (25). Research indicates that gait speed is a dependable measure of muscle function in older adults and is correlated with adverse health outcomes, including disability, falls, and mortality.

Despite the availability of diagnostic tools, no established normative reference exists for diagnosing sarcopenia in the Philippines. A scoping review has highlighted the use of various assessment methods in sarcopenia studies among elderly Filipinos, including the EWGSOP and AWGS criteria, magnetic resonance imaging (MRI), and psoas muscle thickness divided by height (PMTH) (5). These studies have identified potential cut-off values for muscle mass, strength, and performance tailored to the Filipino population. Given the variability in population characteristics across countries, there is an urgent need for studies to establish appropriate cut-off points specific to the Filipino population. This would facilitate the development of local health promotion and sarcopenia management programs. Tee and Tee (26) aimed to establish normative data for muscle mass and physical performance among young Filipinos to enhance the applicability of EWGSOP criteria. Their study provides reference values for normal muscle mass, strength, and physical performance, thereby contributing to more accurate diagnosis and preventive strategies for sarcopenia. The cut-off points specific to Filipinos derived from this study have been cited in subsequent sarcopenia research in the Philippines, including studies on the association between obesity and sarcopenia and sarcopenia with osteoporosis in patients with chronic obstructive pulmonary disease (25, 26, 52). The diagnostic standards are as follows: (I) Low muscle mass is defined as a lean tissue index of <12.50 kg/m^2^ for males and < 8.33 kg/m^2^ for females using the Fresenius Body Composition Monitor. (II) Low muscle strength is defined as grip strength of <24.54 kg for males and < 16.10 kg for females, measured with a JAMAR dynamometer. (III) Low physical performance is assessed using Usual Gait Speed and Timed Get Up and Go. Usual Gait Speed is measured with the 4-m walk test, with values <0.55 m/s for males and < 0.65 m/s for females considered low. For the Timed Get Up and Go, times >8.31 s for males and > 8.74 s for females are considered indicative of low physical performance. Further research is needed to validate these reference values, specifically in diverse Filipino subpopulations, and to optimize diagnostic criteria.

## 4 Treatment

As highlighted in the review by Gay-as et al. (5), most of the existing research on sarcopenia among older Filipinos focuses on determining the prevalence and associated factors of sarcopenia in hospitalized individuals (secondary prevention). Some studies address the development of consensus on the prevention and management of sarcopenia and its associated factors, while few explore educational initiatives on sarcopenia for healthcare professionals. There is a significant gap in research on the treatment of sarcopenia within the Filipino population. Therefore, this review summarizes current global treatment approaches for sarcopenia to offer guidance for managing sarcopenia in the Philippines, with the goal of enabling older individuals to lead healthy and productive lives in accordance with the Philippine government’s vision for its elderly citizens.

### 4.1 Exercise

Physical activity can be an effective intervention for sarcopenia, although there is considerable variability in the types of physical activity (resistance, aerobic, balance, and combinations thereof) that target sarcopenia. Recent studies have shown that resistance exercise is a proven intervention for sarcopenia, improving muscle mass, strength and physical function in older adults (27). Among other things, high-intensity resistance training is more effective than low-intensity resistance training in improving muscle mass and strength in older adults with sarcopenia (27), as well as reducing body fat content and improving grip strength. Aerobic exercise also plays a significant role in the rehabilitation of sarcopenia. Some studies have demonstrated that aerobic exercise, such as walking or bicycling, improves cardiovascular fitness, endurance, and physical function in older adults with sarcopenia (27), as well as reducing body weight and fat mass. The results of a meta-analysis showed that a combination of aerobic exercise and resistance training reduced body fat percentage and increased walking speed in patients with sarcopenia (28). The combination of two or more types of exercise modalities may improve fitness in multiple ways (knee extension strength, gait speed, grip strength, standing chair time, physical performance, etc.) in patients with sarcopenia (29, 53). Current multicomponent exercise programs that combine resistance training, aerobic exercise, balance training, and flexibility training have been shown to be more effective than single-component exercise programs in improving physical functioning and reducing the risk of falls in older adults with sarcopenia (30). There is high-quality evidence that resistance training, aerobic exercise combined with balance training is effective in improving quality of life in patients with sarcopenia (31).

#### 4.1.1 Resistance movement

Resistance training serves as a primary intervention for sarcopenia, recommending a frequency of 2–3 sessions per week, with each session lasting 30–60 min, for a minimum of 12 weeks. This training is structured in three progressive stages: beginner (40–60% of 1 Repetition Maximum, RM), intermediate, and advanced. Initially, participants should engage in beginner training for 1–2 weeks before progressing to intermediate and advanced stages. Resistance should be incrementally increased by 5–10% of 1 RM per session. The intermediate and advanced stages advocate for medium to high-intensity resistance training (60–80% of 1 RM). In the beginner stage, each exercise should be performed 8–10 times per set, with 1–2 sets per session, and a 1 to 2-min rest between sets. The intensity and volume of training should be escalated gradually. Common resistance exercises include the use of dumbbells, sandbags, elastic bands, grip devices, and bodyweight movements such as sit-ups, push-ups, squats, and jumps (32, 33).

#### 4.1.2 Aerobic exercise

Aerobic exercise is characterized by energy generation through aerobic oxidation, where the respiratory system supplies sufficient oxygen to muscles for sustained activity. Heart rate should be monitored to maintain moderate-intensity exercise (50–80% of maximum heart rate), calculated as 220 minus ages. Aerobic exercise enhances cardiopulmonary function, endurance, immunity, and adaptability, complementing resistance training in a positive feedback loop. When performed alone, aerobic exercise should last 30–45 min, at least 3 times per week (17).

#### 4.1.3 Balance training

Balance training aims to improve physical stability and reduce fall risk in individuals with sarcopenia. It includes: (1) Static balance training, where the body maintains a posture, such as the three-step potential balance (tandem, semi-tandem, and feet together standing), and single-leg stands (eyes open or closed, hands on waist or chair back). Each movement should gradually increase from 10 s to 1 to 2 min; (2) Dynamic balance training, focusing on the body’s balance during movement, through sit-to-stand transitions, walking exercises (forward, backward, sideways) (34).

#### 4.1.4 Family training and educational training

This involves home-based rehabilitation to help patients adapt to the family environment, participate in family life, and interact with family members. Under professional guidance, family members act as trainers, conducting rehabilitation that includes disease education, use of rehabilitation equipment, medical exercises, and household activities. Training plans should be tailored to the patient’s abilities, specifying training time, venue, equipment, and content, utilizing everyday items. Situational training, such as daily routines and community activities, should have clear stage goals.

#### 4.1.5 Exercise rehabilitation prescription

Exercise prescription should be personalized based on the FITT principle, considering the patient’s condition. FITT principle includes: (1) Frequency (F): Patients should increase daily physical activity, with at least three exercise sessions per week. (2) Intensity (I): Recommend moderate resistance exercise (<70% of 1 RM) and aerobic exercise (<70% of VO_2_peak), starting with low intensity and gradually increasing. (3) Time (T): The target duration is 30–60 min per session. (4) Type (T): Include aerobic, resistance, and flexibility exercises. During rehabilitation, the patient’s tolerance should be fully assessed to ensure exercise safety (28).

### 4.2 Nutritional interventions

Nutritional interventions play a crucial role in the rehabilitation of sarcopenia, with common supplements including protein, essential vitamins, leucine and its metabolites, and creatine. Protein and vitamin D supplementation, according to currently available studies, have been shown to be effective in improving muscle health in older patients with sarcopenia (35). However, a meta-analysis showed (36) that protein supplementation increased muscle mass but did not enhance muscle strength and physical performance. However, it may have a synergistic effect on muscle mass and strength in older patients with sarcopenia when combined with resistance training (37). Essential amino acids, which cannot be synthesized in the body, and some studies have shown that intake of complete essential amino acids improves the efficiency of energy conversion in the body more than supplementation of complete proteins, with leucine supplementation being effective in increasing muscle mass in patients with sarcopenia and having a weaker effect in muscle strength or physical performance. In muscle exercise, creatine is involved in the process of muscle contraction, and most studies have combined creatine supplementation with resistance exercise for interventions in patients with sarcopenia, which can increase muscle mass and strength, and also creatine has been considered as a preventive modality for elderly patients with sarcopenia (38).

The Chinese Consensus recommends that sarcopenic patients maintain a daily protein intake of 1.2–1.5 g/kg/day, with at least 50% being high-quality protein. For those with severe malnutrition, a minimum daily protein supplementation of 1.5 g/kg/day is advised (39). For instance, an elderly individual weighing 60 kg should consume 90 g of protein daily. Whey protein, abundant in essential amino acids like leucine and easily digestible, can effectively prevent muscle decay and enhance muscle strength when incorporated into the diet at 30–40 grams/day, alongside regular exercise. Protein intake should be evenly distributed across three meals to optimize muscle protein synthesis rates (36). Essential amino acids, particularly leucine, and L-carnitine are recommended for sarcopenic patients as they stimulate protein synthesis and ameliorate frailty, respectively (39, 40). *β*-Hydroxy-β-methylbutyric acid (HMB), a key metabolite in protein regulation, has been shown to have preventive and therapeutic effects on sarcopenia when used in conjunction with resistance training. It is suggested that elderly sarcopenic patients supplement with 3 g of HMB daily, particularly those with prolonged sedentary or bedridden periods (39). A study cited in the Asian Consensus demonstrated that oral nutritional supplements (ONS) containing 1.5–3 grams/day of the leucine derivative HMB, administered in two doses over 180 days, significantly improved nutritional outcomes and reduced the risk of malnutrition in the intervention group compared to the placebo group (40, 41).

### 4.3 Other rehabilitation

In the general population, hormonal imbalances may contribute to the development of sarcopenia, with hormones such as testosterone, growth hormone, insulin, thyroid hormones, and vitamin D potentially affecting its development. Hormone therapies that may improve the physical performance of sarcopenic patients, such as testosterone replacement therapy and selective androgen receptor modulators (SARMs), have been investigated for the treatment of sarcopenia, with promising results in terms of improved muscle mass and strength (42). Vitamin D is known to be associated with muscle strength, muscle size, and physical performance in patients with sarcopenia, but the outcomes of vitamin D replacement therapy remain controversial. Furthermore, it has also been suggested that the development of sarcopenia in patients with chronic kidney disease may be associated with an extended half-life of growth hormone (GH). In addition, it has been suggested that the development of sarcopenia may have a correlation with symptoms of insulin resistance, which may be related to reduced muscle glucose uptake, further impairs intracellular glucose metabolism and thus leads to muscle proteolysis, and may be a new avenue for targeted therapy (43). Gastric starvation hormone, estrogen, cortisol and dehydroepiandrosterone may be other factors in sarcopenia (44).

Targeted pharmacological interventions based on different hormonal pathways of action may alleviate further progression of sarcopenia, such as muscle growth inhibitor inhibitors and activin receptor type IIB (ActRIIB) ligand traps, which are being developed and tested for the treatment of sarcopenia, with potential benefits in terms of increasing muscle mass and strength (45).

Neuromuscular electrical stimulation (NMES) and whole-body vibration (WBV) are novel therapies, and recent research suggests that NMES and WBV may be useful adjuncts to traditional exercise interventions for sarcopenia (46, 47). Smith et al. (48) concluded that the improvements in balance and leg strength with WBV suggest that this physical intervention is beneficial in reducing the risk and incidence of falls. Machado et al. (49) suggest that adaptations to WBV may help offset the loss of muscle strength and mobility associated with age-induced sarcopenia by contributing to an increase in plantar flexor strength and power in older adults. In Paillard’s (50) study, NMES proved effective in improving postural balance function and reducing the risk of falls in older adults with muscle weakness, but there may be adverse effects of frequent interventions.

## 5 Conclusion

The rising prevalence of sarcopenia in the Philippines, driven by the aging population, highlights the urgent need for effective diagnostic, preventive, and treatment strategies. Despite the growing body of research, there remains a significant gap in the application of sarcopenia research to clinical practice and public health initiatives. Establishing localized diagnostic criteria and expanding treatment options are vital steps in tackling this emerging health challenge. Enhanced awareness, research, and implementation of evidence-based practices are essential to improving outcomes for older adults with sarcopenia in the Philippines.
